# Exploring altermagnetism in RuO_2_: from conflicting experiments to emerging consensus

**DOI:** 10.1186/s40580-026-00532-6

**Published:** 2026-01-28

**Authors:** In Hyeok Choi, Seung Gyo Jeong, Bharat Jalan, Jong Seok Lee

**Affiliations:** 1 Department of Physics and Photon Science, Gwangju Institute of Science and Technology (GIST), Gwangju, 61005 Republic of Korea; 2 Department of Chemistry, Massachusetts Institute of Technology, Cambridge, MA 02139 USA; 3 Department of Chemical Engineering and Materials Science, University of Minnesota–Twin Cities, Minneapolis, MN 55455 USA

**Keywords:** Altermagnet, Spintronics, Strain-engineering

## Abstract

**Abstract:**

Altermagnetism has recently emerged as a new class of magnetic order that combines the advantages of both ferromagnets and antiferromagnets. The compensated antiparallel spin structure, in combination with crystallographic rotational symmetry, gives rise to distinct magnetic properties, opening new opportunities for next-generation spintronic applications. In this review, we introduce a variety of experimental approaches—including electronic, optical, and particle-based spectroscopies—used to probe theoretically suggested altermagnetism. In particular, we review recent studies on the altermagnetic candidate RuO_2_, whose magnetic ground state remains under debate with conflicting experimental results, organizing the discussion according to the experimental techniques. Furthermore, we highlight recent findings on fully strained RuO_2_ thin films that emphasize the critical role of strain in the emergence of altermagnetism. We believe that this review will provide not only practical guidelines for investigating altermagnetic systems but also valuable insights toward reaching consensus on the ongoing controversies surrounding RuO_2_’s altermagnetism.

**Graphical Abstract:**

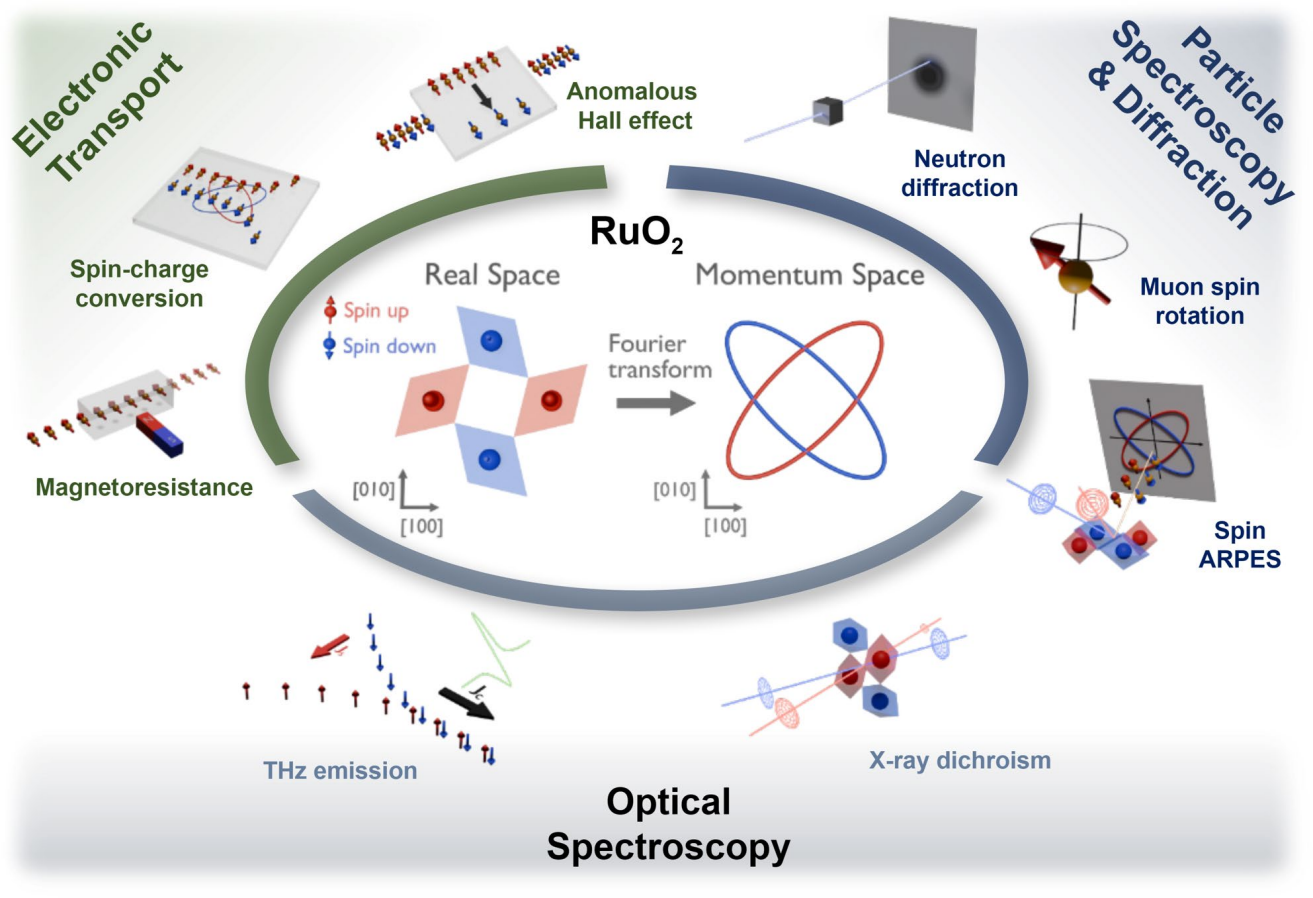

## Introduction

Compensated spins under rotational crystal symmetries break the translation symmetry, resulting in momentum-dependent non-trivial spin splitting in the reciprocal space [1, 2]. This unique spin-split band structure with antiferromagnetic (AFM) spin configuration, i.e., altermagnetic (AM) state, introduces a new paradigm for writing and reading of spin information with higher storage density and high stability even under external magnetic fields. Moreover, AMs combine the advantages of both AFM and ferromagnets (FMs)—such as easy access to and switching of spin polarization with THz speed—providing high controllability and compatibility with existing spintronic devices. These novel properties make AMs promising candidates for next-generation spintronic applications in sensing, storage, and computing.

**Fig. 1:**
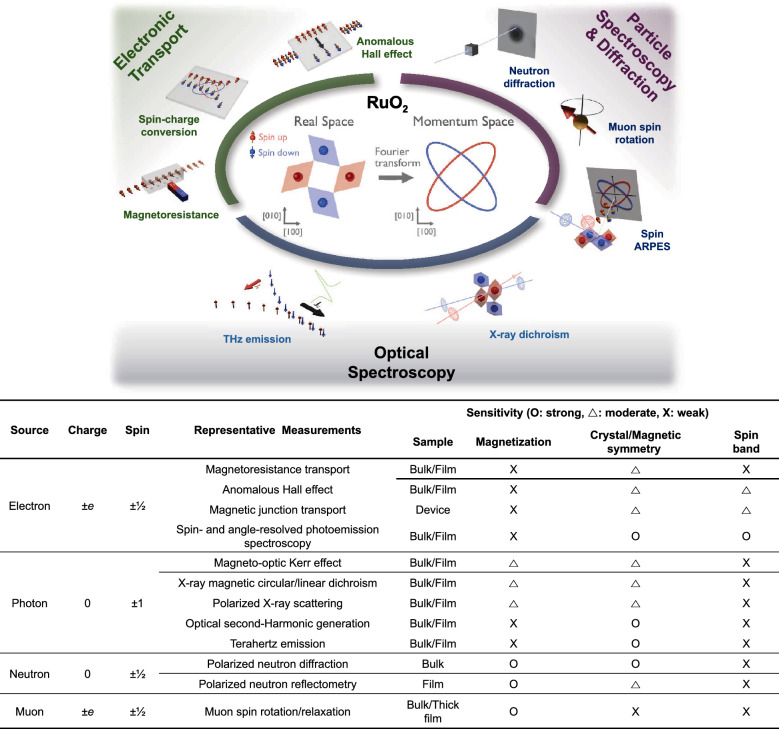
Summary of representative experimental probes for investigating AM. Each probe is categorized by its source particle, intrinsic charge, and spin, along with typical measurement techniques and the relevant sample form. The relative sensitivity of each method to magnetization, crystal/magnetic symmetry, and spin-band structure is indicated as O (strong), Δ (moderate), or × (weak).

To investigate the characteristic magnetic properties in AMs, a wide range of experimental probes and techniques have been employed. Figure 1 summarizes the sensitivities of massive-particle- (neutron or muon), electron-, and photon-based measurements to key properties of AMs, including magnetization, crystal and magnetic symmetry, and spin-split electronic band structures. Neutron [3–6] and muon [4, 7, 8] probes directly couple to magnetic moments, providing spatially-resolved and dynamic insights into intrinsic magnetization and magnetic depth profiles [9]. Furthermore, spin- and angle-resolved photoemission spectroscopy (spin-ARPES) [10–13] enables direct probing of non-relativistic spin-split band structures in AMs. Electron-based transport measurements, such as magnetoresistance and the anomalous Hall effect [14–21], reveal signatures of spin-polarized carriers and the symmetry of spin bands, while device-level studies, including magnetic tunnel junctions [22–24], spin-splitting torque [25–33], and spin Hall measurements [33–41], enable the detection of interfacial magnetism and quantum spin transport, paving the way for spintronic applications. Photon-based techniques, including the optical spectroscopy [42], THz emission spectroscopy [43–46], magneto-optic Kerr effect [47, 48], X-ray magnetic circular/linear dichroism [30, 49–51], X-ray diffraction [52–55], and optical second-harmonic generation [56], probe crystal and magnetic symmetry, spin-split bands, and spin-dependent optical transition. Due to the high sensitivity to surfaces or interfaces, they can enable the investigation of AMs stabilized in ultrathin films.

**Fig. 2:**
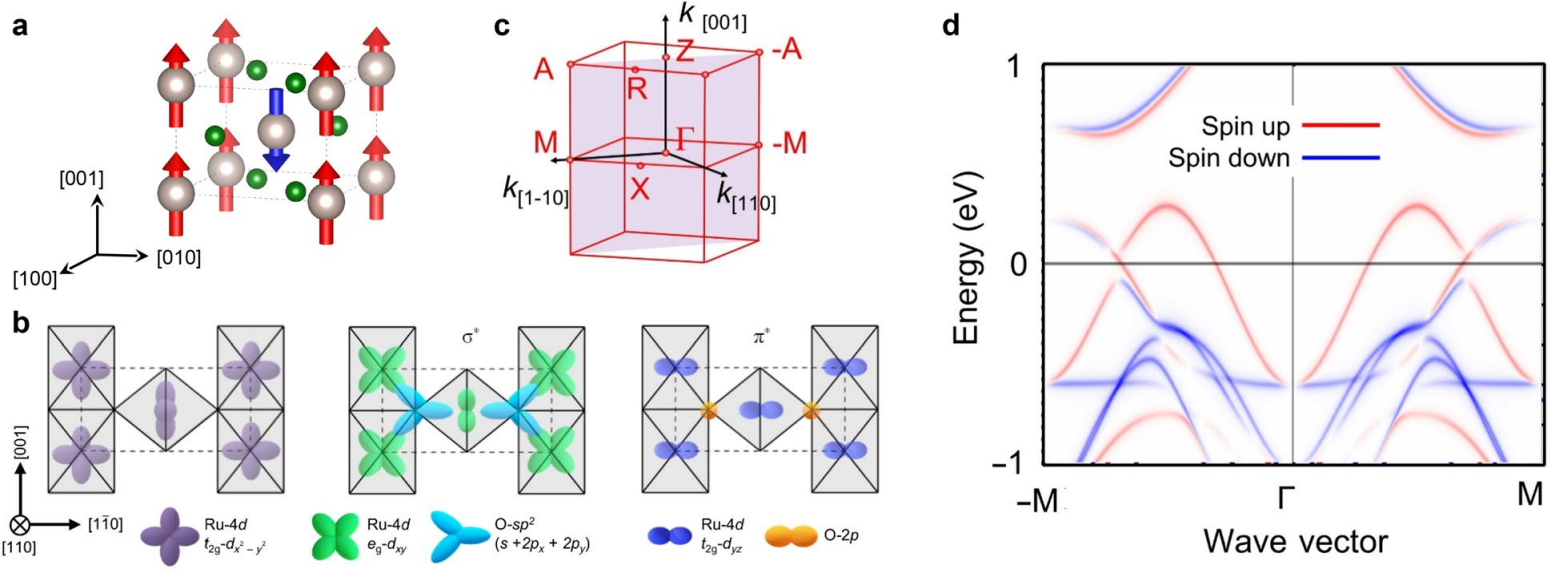
Structural, orbital, and spin characteristics of RuO_2_. **a** Schematic of P4_2_/*mnm* rutile structure of RuO_2_ with the collinear antiferromagnetic order parameter along the [001] direction. **b** Hybridized orbital structure of Ru 4d and O 2p states. Reprinted from Ref. [62] (Licensed under CC BY-NC 4.0.). **c** Brillouin zone of rutile RuO_2_ and **d** density functional theory calculation of spin splitting band in altermagnetic RuO_2_. Reprinted from Ref. [10] (Licensed under CC BY 4.0.).

**Fig. 3:**
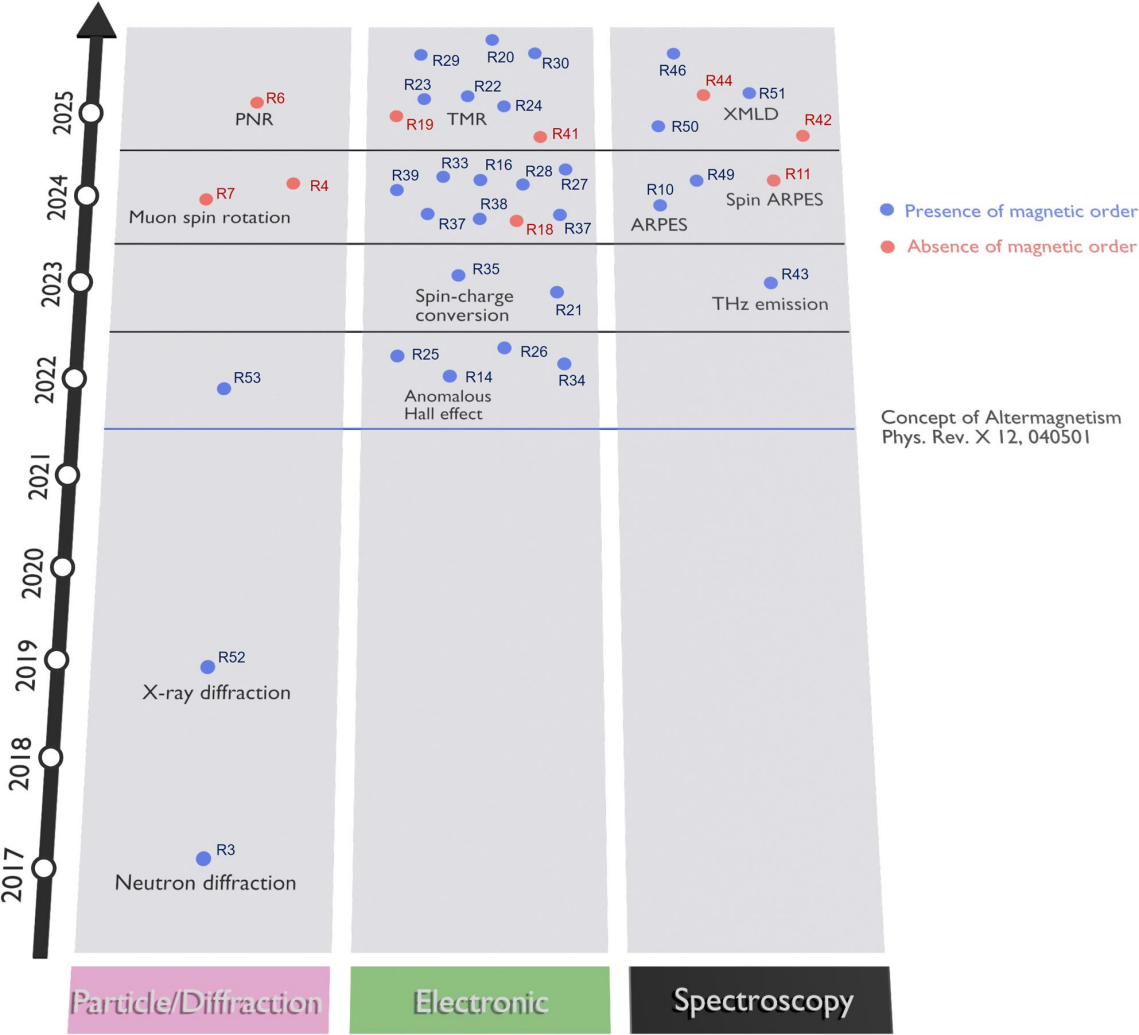
Timeline of publications investigating magnetic or AM order in RuO_2_, categorized into particle/diffraction, electronic, spectroscopy-based methods. Blue and red scatters are papers suggesting the presence and the absence of magnetic order in RuO_2_, respectively. The corresponding references (R#) are categorized according to their year of publication.

These complementary techniques form a powerful and synergistic framework for revealing the magnetic ground state and elucidating the interplay among spin, charge, and symmetry in correlated AM systems. RuO_2_ was the first material theoretically proposed as an AM candidate [2], predicted to exhibit promising AM functionality at room temperature. Bulk RuO_2_ was reported to have the tetragonal 4/*mmm* structural and 4′/*mm*′*m* magnetic point group [2], where the antiferromagnetic Néel vector is along the [001] axis (Fig. 2a). RuO_2_ exhibits high electrical conductivity [57] and has attracted significant attention for applications in electrochemical catalysis [58], thermometry [59], integrated circuits [60], and plasmonics [61]. The Ru-4*d* orbitals of RuO_2_ split into three *t*_2g_ states and two *e*_g_ states, with the electronic states at the Fermi level predominantly derived from the Ru *t*_2g_ ​states, giving rise to the metallic conductivity of RuO_2_ [62] (Fig. 2b). Theoretical calculations further predicted substantial *d*-wave altermagnetic spin splitting of approximately 1.4 eV along the *M*-direction in the *k*-space, implying strong potential for spintronic applications [2, 10] (Fig. 2c, d). However, its magnetic ground state remains under debate due to conflicting experimental results. Figure 3 summarizes published studies reporting either the presence or absence of magnetic and/or AM states in RuO_2_, represented by blue and red markers, respectively. The data are categorized according to the measurement technique—electronic, optical spectroscopy, and particle spectroscopy or X-ray diffraction. Since the introduction of the AM concept in 2022 [1, 2], the number of related publications has grown rapidly. In the same year, the AM state was experimentally reported in RuO_2_ thin films through anomalous Hall effect with 50 T of magnetic fields [14]. However, later muon spin rotation/relaxation (μSR) [4, 7] and density functional theory (DFT) [15] studies challenged this finding, arguing that stoichiometric RuO_2_ does not exhibit an AM state. As a result, reports indicating the absence of magnetic order—particularly in bulk RuO_2_—have become increasingly common, underscoring the continuing debate over its true magnetic ground state [1, 2, 4, 7, 14, 15].

In this review, we begin by summarizing recent experimental observations in identifying the structural and magnetic structure of RuO_2_ for both bulk and thin film samples using particle-based spectroscopies. We also discuss transport-based studies that have provided compelling evidence for spin-polarized conduction in RuO_2_ thin films. We then examine controversies in the spectroscopic measurements, including spin-ARPES studies, highlighting the conflicting reports and ongoing debates concerning spin-split band structures in RuO_2_. Next, we discuss the recent studies highlighting the key role of epitaxial strain/dimensionality in stabilizing the AM state in ultrathin RuO_2_ films. Finally, we offer a perspective on the future of AM research and its potential technological impact. Advancing high-quality thin-film and bulk synthesis while understanding the role of defects (point defect, line defect, and surface-related defects), refining interface engineering, and developing cutting-edge characterization techniques will be key in revealing the microscopic origins of AM and enabling its integration into next-generation spintronic and quantum technologies.

## On-going controversies: altermagnetism in RuO_2_

The magnetic ground state of RuO_2_ remains under debate across various experimental techniques and samples synthesized by different growth methods. These suggest that the discrepancies originate not only from measurement methodologies but also from variations in growth conditions, dimensionality, chemical states, and epitaxial strain. In this section, we summarize several conflicting experimental findings obtained using particle-based, electron-based, and optical-based spectroscopic probes.

### Controversies in particle-based spectroscopy studies

Particle-based spectroscopy techniques employing electrons, neutrons, and muons offer exceptionally high sensitivity to magnetic responses in materials. Polarized neutron diffraction and reflectometry provide unique access to the spatial distribution of magnetic moments and magnetic depth profiles in bulk and thin-film systems, respectively. μSR measurements deliver local information on internal magnetic fields and dynamic spin fluctuations, making them indispensable for detecting magnetism in systems lacking long-range order. Although these particle-based probes typically require bulk or relatively thick samples, their sensitivity to intrinsic magnetization is remarkably high.

**Fig. 4:**
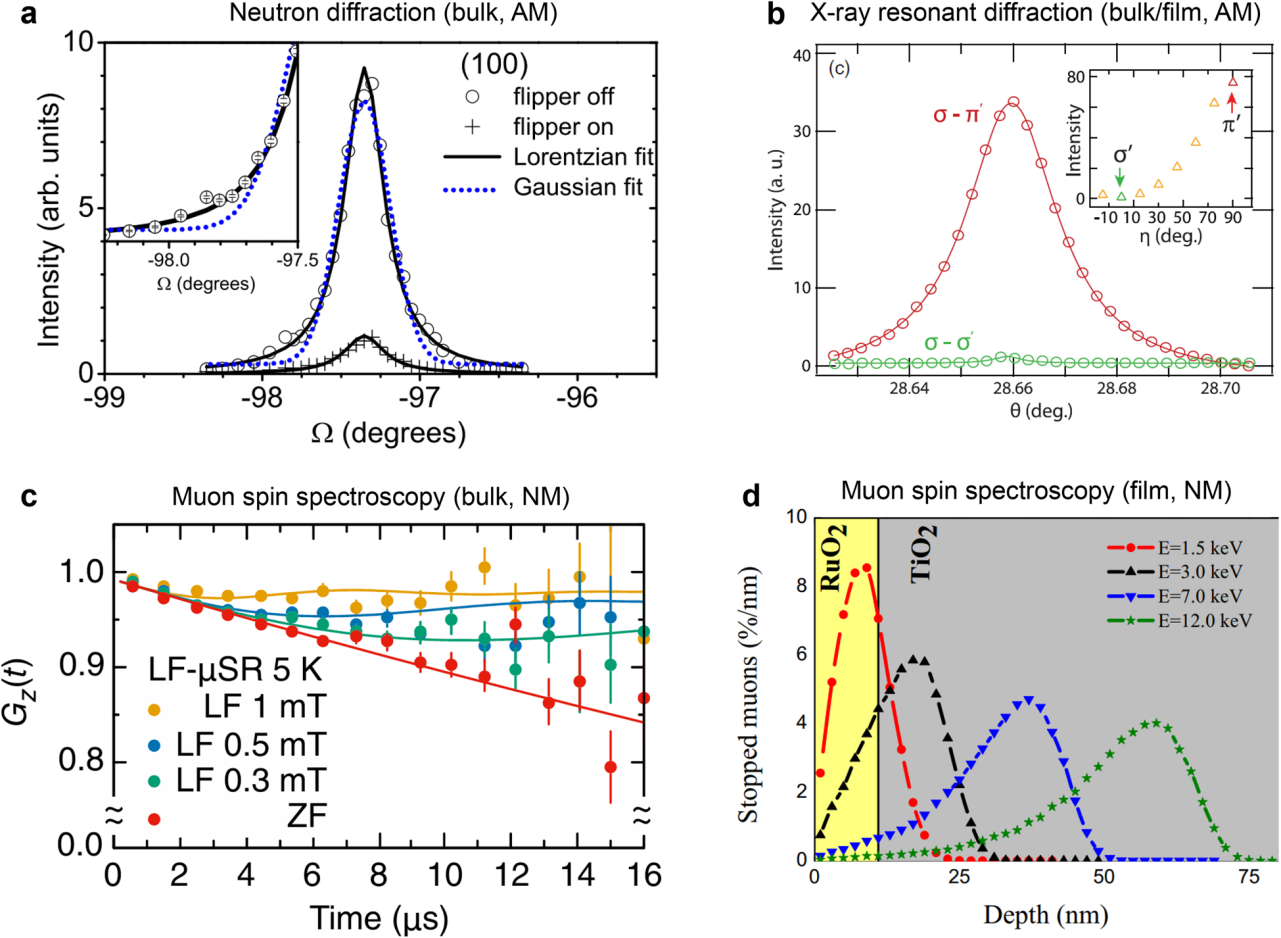
Neutron- and muon-based probing approaches to measure magnetic response in RuO_2_. **a** The (100) Bragg reflections of RuO_2_ bulk measured using polarized neutron diffraction, suggesting AFM order. Reprinted with permission from Ref. [3] (Copyright © 2017 by the American Physical Society). **b** AFM ordering from polarization dependence of the (100) Bragg reflections of RuO_2_ using resonant X-ray scattering. Reprinted with permission from Ref. [52] (Copyright © 2019 by the American Physical Society). **c** Time-dependent Muon spin spectrum of RuO_2_ bulk with different magnetic fields. Reprinted with permission from Ref. [7] (Copyright © 2024 by the American Physical Society). **d** Depth profile of stopped muons signals of RuO_2_ (110) epitaxial films. Reprinted from Ref. [4] (Licensed under CC BY 4.0.).

Berlijn et al*.* first reported the observation of the (100) Bragg diffraction peak in bulk RuO_2_, which reflects both the anisotropic tetragonal crystal structure and AFM order along the [001] direction [3] (Fig. 4a). Spin-polarized neutron scattering measurements revealed a small, ordered moment of approximately 0.05 μ_B_ at room temperature in bulk RuO_2_ [3]. Subsequent resonant X-ray scattering experiments further confirmed the (100) Bragg diffraction peak in both bulk and thick-film RuO_2_ samples [52] (Fig. 4b), although distinguishing between structural and magnetic contributions to this reflection remains challenging [54, 55].

Contrary to these early observations, recent μSR studies on both RuO_2_ bulk and film by Hiraishi et al. provided no clear evidence of magnetic ordering, indicating a predominantly non-magnetic ground state [4, 7] (Fig. 4c, d). They estimated a very weak internal field corresponding to an effective moment of about 4.8 × 10^–4^ μ_B_ in bulk RuO_2_ [7] (Fig. 4c), much smaller than that inferred from neutron scattering [3]. Similarly, μSR measurements on 12 nm thick RuO_2_ thin films grown on TiO_2_ (110) substrates using pulsed laser deposition (PLD) revealed slightly enhanced muon stopping signals compared with bulk RuO_2_ and the TiO_2_ substrate (Fig. 4d), although the estimated magnetic moment remains extremely small (~ 7.5 × 10^–4^ μ_B_) [4]. On the other hand, it is interesting to note that the low-energy μSR study reported increased muon asymmetry near the film surface [8]. This could be understood as the possible emergence of weak magnetic ordering in the near-surface region.

**Fig. 5:**
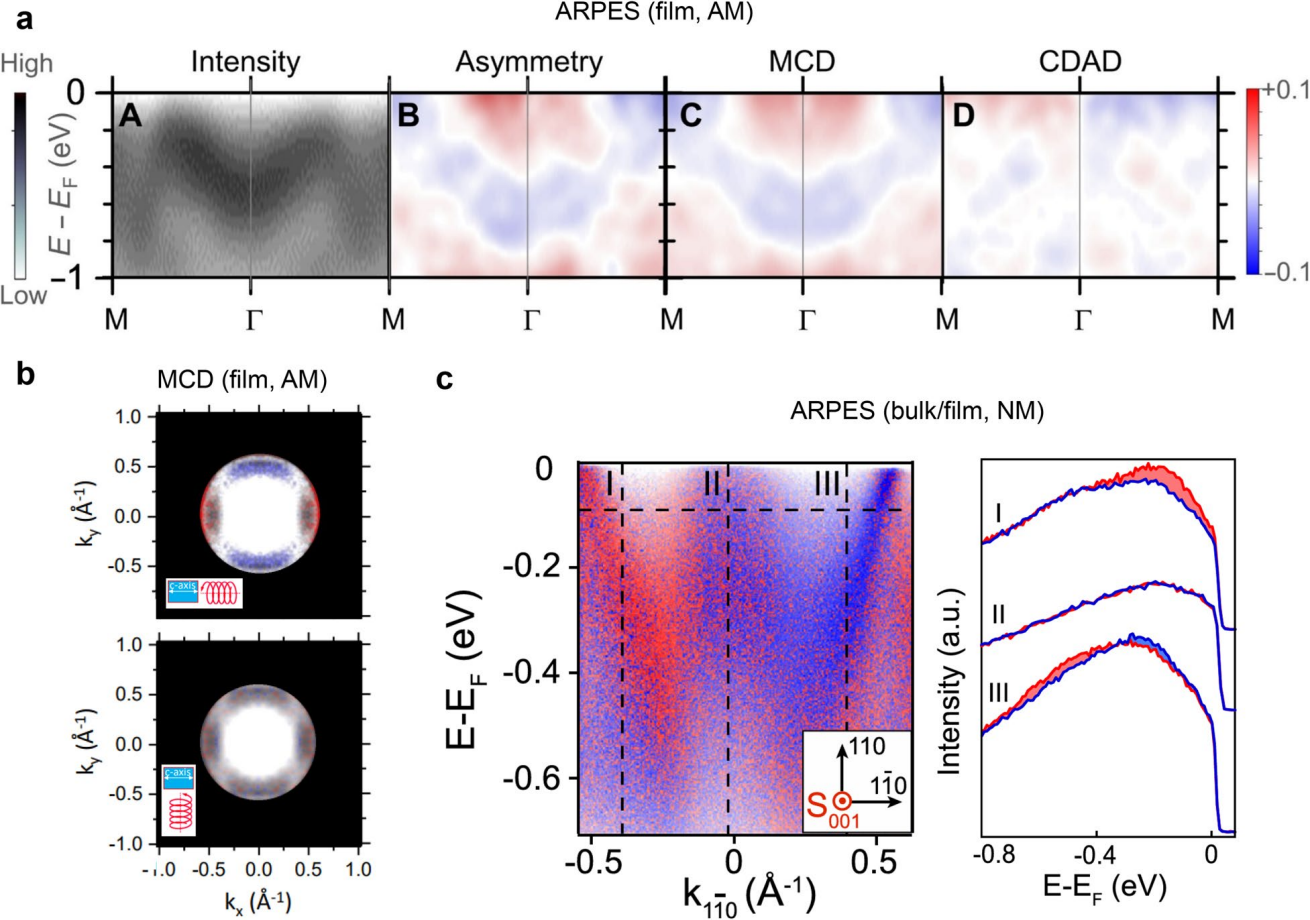
Spin splitting in RuO_2_ measured by ARPES. **a** ARPES with magnetic circular dichroism (MCD) results of epitaxial RuO_2_/TiO_2_ (110) films, and **b** MCD results on the Fermi surface of RuO_2_/TiO_2_ (110) films. Reprinted from Ref. [10] (Licensed under CC BY 4.0.). **c** Rashba-like spin splitting in RuO_2_ bulk. Reprinted with permission from Ref. [11] (Copyright © 2024 by the American Physical Society). AM and NM indicate reporting magnetic and non-magnetic states in RuO_2_, respectively.

Spin-resolved ARPES provides a direct means to demonstrate spin-split electronic bands, which are regarded as a smoking gun for revealing the AM nature of RuO_2_. We note, however, that the existence of the AM state in RuO_2_ is also under debate in light of recent spin ARPES results. Fedchenko et al. reported time-reversal symmetry breaking in the band structure of RuO_2_ using ARPES with magnetic circular dichroism (MCD) [10], as shown in Fig. 5a. They quantitatively concluded the existence of spin-split bands and AM order in 34 nm RuO_2_/TiO_2_ (110) films. They also obtained the spin-splitting Fermi surfaces using circularly polarized ultraviolet light incident along the [001] and the [1$\overline{1}$0] direction (Fig. 5b), supporting AM order in RuO_2_. On the other hand, Liu et al. argued the presence of spin splitting in in both bulk and thick-film RuO_2_ samples using spin-resolved ARPES, whereas it does not break the mirror symmetry along the [001] direction, which could be interpreted not as an AM origin but rather as a Rashba-like spin splitting arising from inversion symmetry breaking [11] (Fig. 5c). This contrasting result highlights the need for a more detailed classification of the mechanisms responsible for mirror symmetry breaking in reciprocal space.

### Controversies in electron-based transport studies

**Fig. 6:**
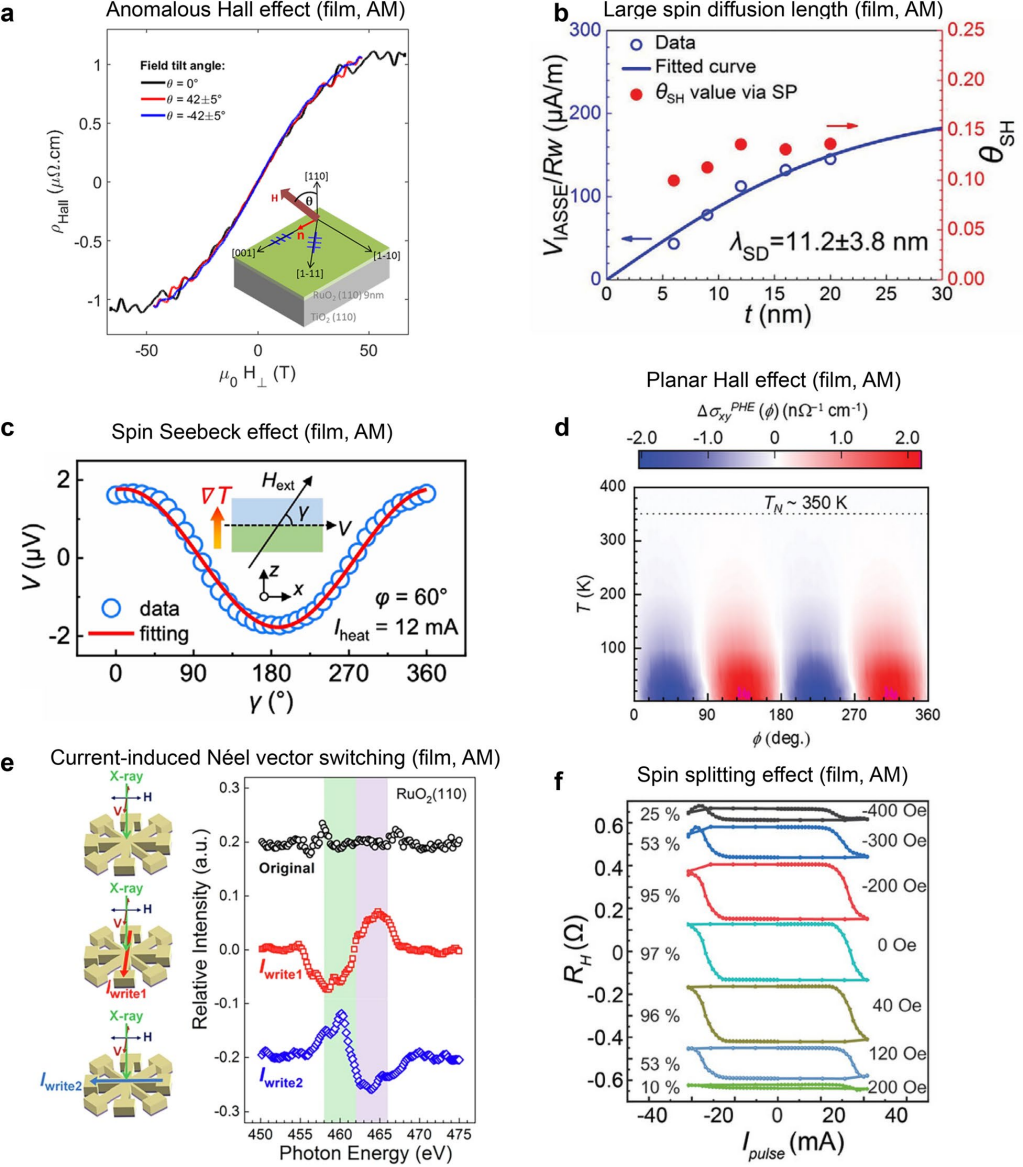
Electrical transport results in RuO_2_ thin films. **a** High magnetic field-dependent anomalous Hall effect with varing magnetic field directions. Reprinted from Ref. [21] (Licensed under CC 4.0). **b** Large spin diffusion length of RuO_2_ (100) measured by thickness dependence of the (AM) spin Hall effect. Reprinted with permission from Ref. [37] (Copyright © 2024 Wiley–VCH GmbH). **c** Spin Seebeck effect depending on the external magnetic direction. Reprinted with permission from Ref. [35] (Copyright © 2023 by the American Physical Society). **d** Temperature-dependent planar Hall effect of epitaxial RuO_2_/TiO_2_ (110) films. Reprinted with permission from Ref. [16] (Copyright © 2024 Wiley–VCH GmbH). **e**, **f** Current-induced magnetic vector switching using **e** XMLD and **f** spin Hall effect device. Reprinted with permission from Ref. [30] (Licensed under CC BY-NC-ND 4.0.) and [29] (Copyright © 2025 Wiley–VCH GmbH), respectively. AM and NM indicate reporting magnetic and non-magnetic states in RuO_2_, respectively.

Electron-based transport measurements provide a signature of spin-polarized electron carriers from the AM spin bands, and there have been a large number of reports supporting the presence of AM in RuO_2_ thin films. Figure 6a shows the anomalous Hall effect (AHE) with magnetic fields exceeding 50 T in a 9 nm RuO_2_/TiO_2_ (110) film grown via PLD, which was attributed to field-induced weak magnetization from in-plane [001] (expected magnetic easy axis) to the out-of-plane [110] directions [21]. Recent studies on hybrid molecular beam epitaxy (MBE)-grown films have shown an AHE at significantly lower magnetic fields (< 6 T) in ultrathin (< 4 nm) RuO_2_/TiO_2_ (110) films, attributed to the presence of epitaxial strain [20]. In contrast, relaxed films exhibit no such effect, highlighting the critical role of epitaxial strain in inducing uncompensated magnetic order in fully strained RuO_2_ films.

Figure 6b shows the spin diffusion length (*λ*_SD_) extracted from nonlocal spin transport measurements in a Py (8 nm)/Al (2 nm)/RuO_2_ (6 nm) trilayer on *r*-Al_2_O_3_ [37]. This study reveals a sizable *λ*_SD_ of ~ 11 nm, suggesting a practical advantage of RuO_2_ for spintronic applications. Bai et al*.* reported spin-to-charge conversion using spin Seebeck effect measurement in Py/RuO_2_ (12 nm) bilayer on *r*-Al_2_O_3_ [35] by measuring a sinusoidal Seebeck voltage as a function of magnetic field angle (γ) (Fig. 6c). Song et al*.* observed a planar Hall effect in RuO_2_/TiO_2_ films [16], which appeared only below 350 K, and attributed it to the emergence of AM ordering (Fig. 6d). Zhang et al. demonstrated current-induced modulation of magnetic order in RuO_2_ films; the AFM vector, traced with X-ray magnetic linear dichroism (XMLD) measurements, could be successfully reoriented by controlling the current direction [30] (Fig. 6e). Furthermore, Zhuoyi et al*.* demonstrated 100% field-free spin–orbit torque (SOT) switching in Ta/[Co/Pt]_2_/RuO_2_(101) heterostructures [29] (Fig. 6f).

The pronounced spin-charge conversion in RuO_2_ thin films enables a realization of spin-dependent tunneling magnetoresistance (TMR) devices. When an ultrathin insulating layer is sandwiched between two conducting layers, electric current can flow across the junction via quantum tunneling. If the conducting layers have AFM or AM order parameters, the junction resistance can be controlled by manipulating spin states that modify the electronic band structure. Noh et al. demonstrated recently the spin-dependent TMR in CoFeB/TiO_2_/RuO_2_ (110) tunnel junctions, with a TMR ratio of up to 5% at 10 K [23]. The junction resistance is controlled by the spin orientation of the CoFeB FM layer, providing evidence for altermagnetic band splitting in RuO_2_ thin films. Furthermore, Xu et al. reported the giant tunneling anisotropy magnetoresistance (TAMR) ratio up to 60% in RuO_2_ (100)/MgO/RuO_2_ (100) tunnel junctions [22]. They revealed that the junction resistance exhibits the magnetic field dependence, which can be elucidated by spin-flop effect in RuO_2_ layers.

In contrast, magneto-transport measurements on bulk RuO_2_ reported non-magnetic behavior [7, 8]. Both the magnetoresistance and Hall resistivity were explained by the Lorentz force in RuO_2_ single crystals. In particular, the observed nonlinear Hall effect in single crystals was attributed to the ordinary Hall effect within a multicarrier model [18], making it difficult to distinguish from the AHE reported in RuO_2_ thin films. Notably, however, fully strained ultrathin RuO_2_ films have exhibited the AHE only below a critical thickness [20], and even under an in-plane magnetic field [20], weakening the argument in favor of the multiple carrier model. Collectively, these findings suggest that the materials differ between different labs, while highlighting the important role of defect, epitaxial strain in stabilizing the magnetic/AM phase in RuO_2_ thin films [25–40]. These results also underscore the potential of epitaxially strained RuO_2_ thin films as a promising platform for energy-efficient spintronic applications.

### Controversies in optical spectroscopy studies—THz emission

**Fig. 7:**
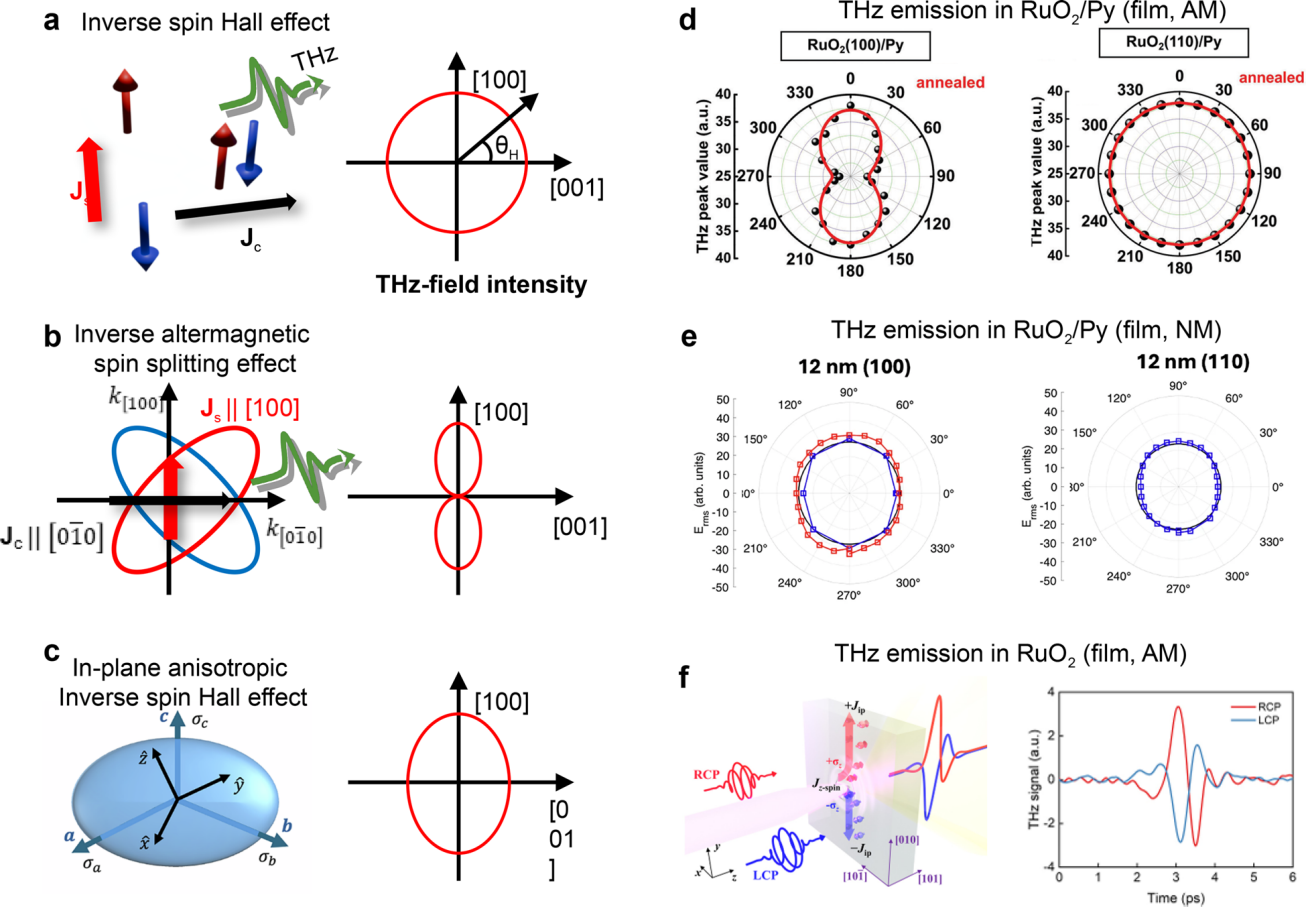
Terahertz (THz) emission spectroscopy on RuO_2_/Permalloy (Py) heterostructures to investigate spin-charge conversion. **a**–**c** Possible THz emission mechanisms in RuO_2_ and their expected magnetic-field-angle (θ_H_) dependent THz-field-intensity polar patterns. Reprinted from Ref. [44] (Licensed under CC BY-NC-ND 4.0.). **d** θ_H_-dependent THz-field-intensity polar patterns of RuO_2_ (110)/Py (left) and RuO_2_ (100)/Py (right) deposited on MgO and YSZ substrates, respectively. To achieve a single domain state, both samples were annealed under magnetic field. Reprinted with permission from Ref. [43] (Copyright © 2023 Wiley–VCH GmbH). **e** Another reported θ_H_-dependent THz-field-intensity polar patterns of RuO_2_ (110)/Py (left) and RuO_2_ (100)/Py (right) deposited on TiO_2_ substrates. Adapted from Ref. [44] (Licensed under CC BY-NC-ND 4.0.). **f** THz emission from RuO_2_ (101) film generated by circularly-polarized optical pulse. Reprinted with permission from Ref. [46] (Copyright © 2025 by the American Physical Society). AM and NM indicate reporting magnetic and non-magnetic states in RuO_2_, respectively.

The spin-charge conversion using AM can be further investigated by the magnetic-field-dependent terahertz (THz) emission technique using metal/permalloy (Py) heterostructures. Photo-induced spin current in the Py layer propagates into the metal layers, generating a THz pulse through the inverse spin Hall effect (ISHE) and the inverse AM spin-splitting effect (IASSE), as shown in Fig. 7a, b. IASSE depends on crystallographic orientation, resulting in anisotropic θ_H_-dependent THz-field-intensity polar patterns. For RuO_2_, an anisotropic pattern is expected in (100)-oriented films but absent in (110)-oriented ones.

A series of studies on the IASSE in RuO_2_ have been reported recently, but they have also generated considerable controversy. The aforementioned prediction of the spin-charge conversion in RuO_2_-based heterostructure was confirmed by Liu et al., who observed an anisotropic pattern in RuO_2_ (100)/Py and an isotropic pattern in RuO_2_ (110)/Py [43] (Fig. 7d). Plouff et al*.*, however, argued that the anisotropic THz emission could be attributed to electronic anisotropic conductivity (EAC) in anisotropic RuO_2_ rather than the IASSE effect [44] (Fig. 7c). As shown in Fig. 7e, they observed anisotropic θ_H_-dependent polar patterns for both (100)- and (110)-oriented RuO_2_ (blue lines), which can be explained by EAC. Furthermore, Jechumtál et al*.* conducted a quantitative analysis of THz emission from RuO_2_/Py by fully considering possible origins [45], and they reported that the spin Hall angle from IASSE was 2 × 10^−4^, much smaller than the theoretical value. Despite the contradicting results in Py/RuO_2_ heterostructures, Cao et al*.* reported IASSE in RuO_2_ (101) films without Py layers using circularly polarized optical pulses, inducing *z*-polarized spin current [46] (Fig. 7f). They observed clear THz emission signals depending on the helicity of light, implying an IASSE origin that shows spin polarization dependence. These conflicting results from THz emission experiments on RuO_2_ imply that the magnetic properties of RuO_2_ might be influenced by defects, strain, or dimensionality effects, so more careful characterization of sample quality and strain state is required for the study of RuO_2_.

## Strain-induced stabilization of altermagnetism in RuO_2_

Recent theoretical studies suggest that the electronic band structure of RuO_2_ is strongly influenced by doping, interface effects, and strain, all of which can impact its magnetic ground state [15, 20, 63]. Notably, strain has been shown to induce a magnetic ground state in ultrathin RuO_2_ films [13, 20, 48, 56, 63]. In this section, we review several studies on fully strained RuO_2_ ultrathin films, highlighting the strain-induced stabilization of the AM state.

### Strain-induced evolution of the electronic structure of RuO_2_ films

**Fig. 8:**
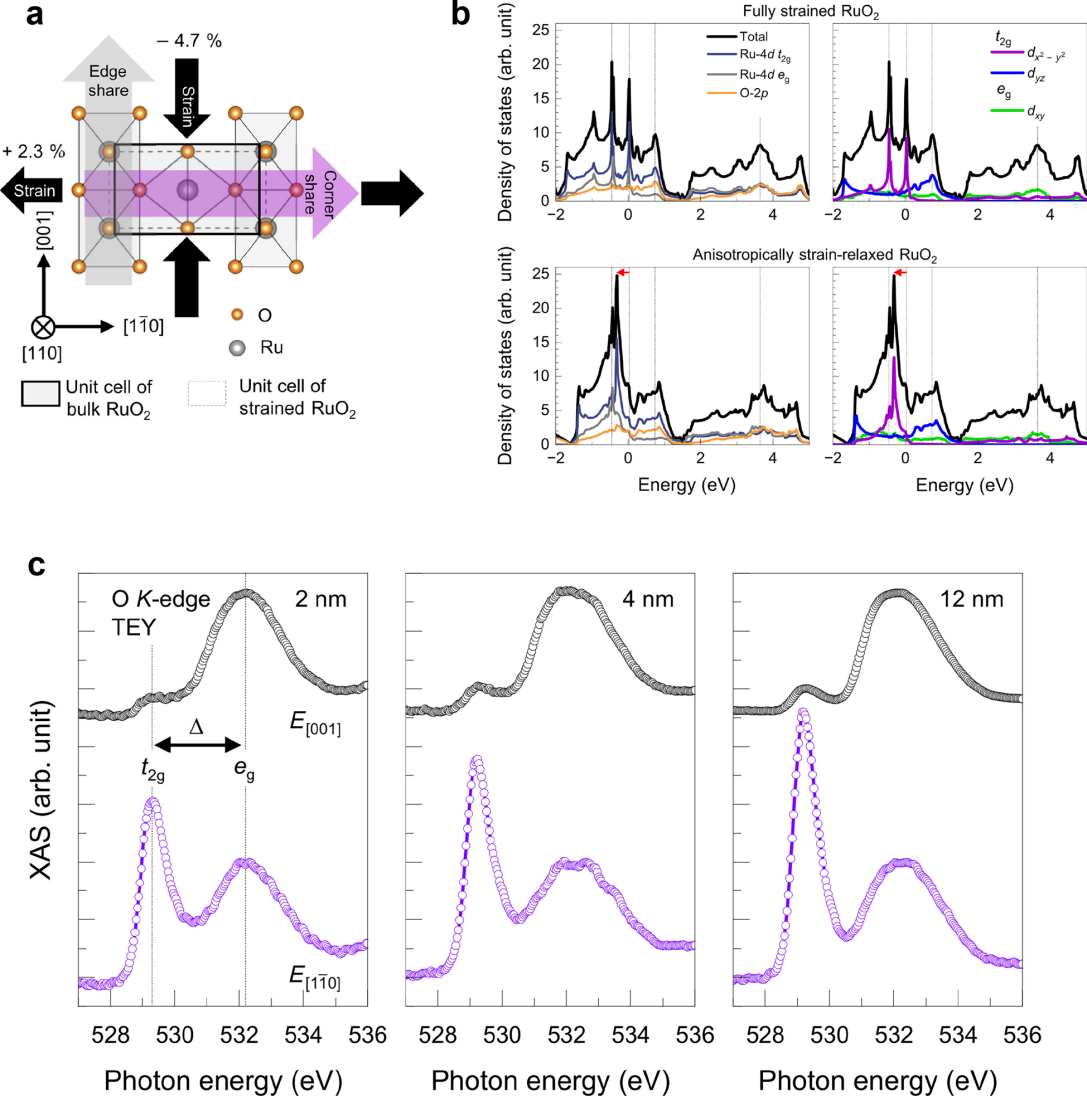
Stain-modified electronic band structure in RuO_2_/TiO_2_ (110) heterostructures. **a** Anisotropically strained RuO_2_ due to lattice mismatch between RuO_2_ film and TiO_2_ substrate. **b** Density functional theory (DFT) calculation on fully strained (top panel) and anisotropically strain-relaxed RuO_2_. **c** Polarization-dependent oxygen K-edge X-ray absorption spectroscopy (XAS) obtained in total electron yield (TEY) mode for 2 nm, 4 nm, and 12 nm RuO_2_/TiO_2_ (110) films. Reprinted from Ref. [62] (Licensed under CC BY-NC 4.0.).

In 2025, a large modulation of the electronic band structure in RuO_2_/TiO_2_ heterostructures under strain was reported, suggesting a potential route to AM RuO_2_ [62]. In the fully strained state, RuO_2_ (110) films on TiO_2_ substrates exhibit a compressive strain of −4.7% along the [001] direction and a tensile strain of + 2.3% along the [$1\overline{1 }0$] direction (Fig. 8a). Due to this strong anisotropic strain effect, hybrid MBE-grown RuO_2_ thin films exhibit anisotropic strain relaxation above 4 nm, occurring only along the [001] direction, which show significantly different properties from those of the bulk. Figure 8b shows DFT calculations for fully strained (top panel) and anisotropically strain-relaxed (bottom panel) RuO_2_, revealing distinct band structures. Near the Fermi level, several momentum-selective hybridized states are found between Ru 4d_*yz*_ (*t*_2g_) and O 2p_*y*_, and between Ru 4d_*xy*_ and O 2p_*x*_ orbitals, while Ru 4d_*x*_^2^_−__*y*_^2^ remains non-hybridized. Consistent with this theoretical prediction, electronic structures verified by oxygen K-edge X-ray absorption spectroscopy (XAS) confirm that strain dramatically modifies the strength of orbital hybridization [62]. Figure 8c displays XAS spectra measured in total electron yield (TEY) mode for RuO_2_ (110) films of various thicknesses, obtained with probe polarizations along [001] (black) and [$1\overline{1 }0$] (purple) directions. Clear *t*_2g_ and *e*_g_ peaks are observed for all thicknesses, exhibiting polarization dependence due to the anisotropic orbital states. Moreover, upon strain relaxation (> 4 nm), the *t*_2g_ peak intensity decreases markedly, demonstrating that the electronic structure of RuO_2_ can be effectively tuned through strain engineering. This strain-modulated electronic structure can provide an opportunity to control the magnetic state in RuO_2_ films.

### Observation of strain-induced magnetic state of hybrid MBE-grown RuO_2_ films

**Fig. 9:**
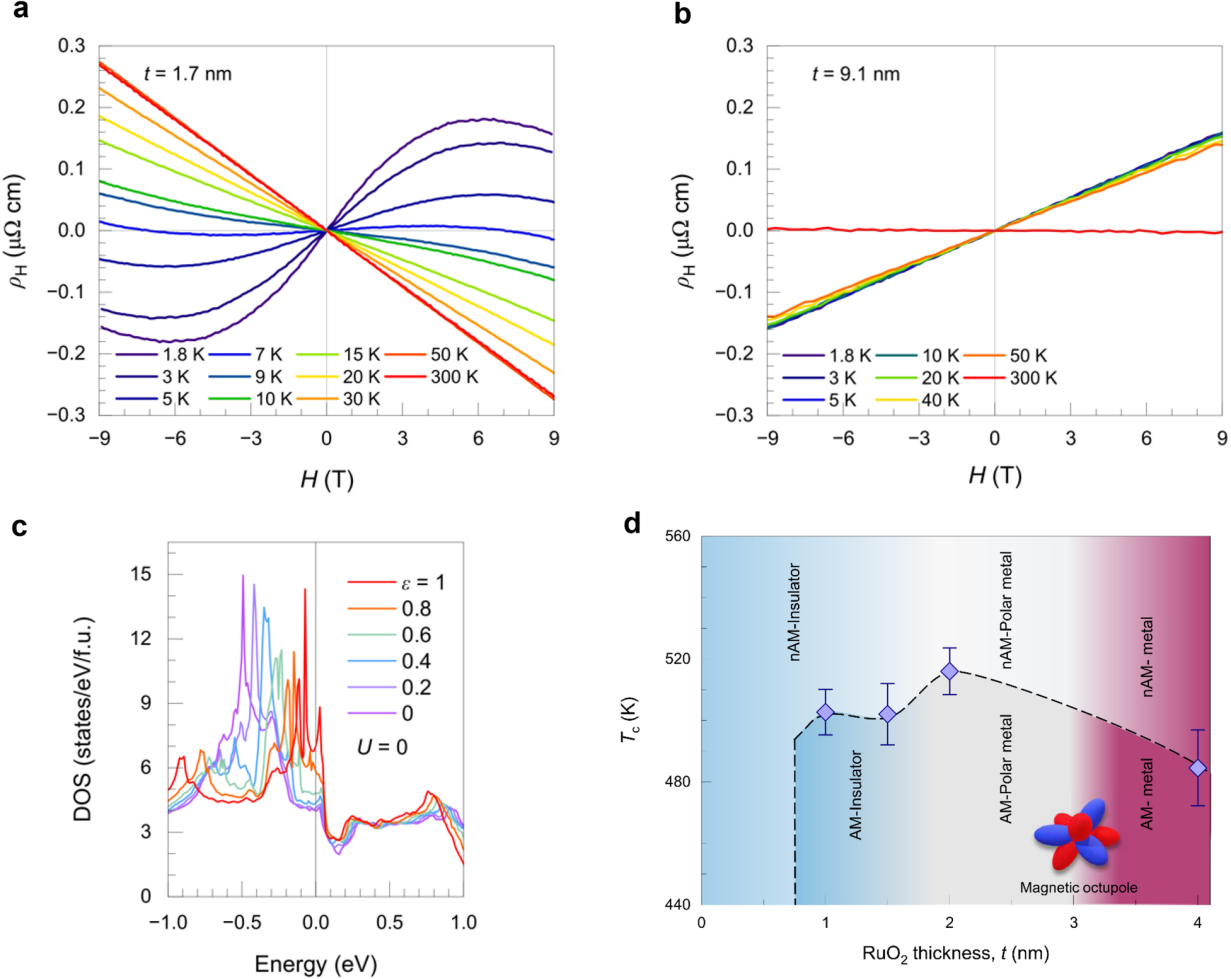
Strain-controlled magnetic state in RuO_2_ (110)/TiO_2_ heterostructures. **a**, **b** Temperature-dependent magneto-transport results for **a** 1.7 nm and **b** 9.1 nm RuO_2_ (110)/TiO_2_. **c** DFT calculation results of the electronic density of states obtained at several strain ε under a zero Hubbard *U* value. Here, ε = 1 is fully strained state. **d** Phase diagram of hybrid MBE-grown RuO_2_ thin films. Reprinted from Ref. [20] (Licensed under CC BY-NC-ND 4.0.) and [56].

Along with the modification of electronic structures in hybrid MBE-grown ultrathin RuO_2_ films, a strain-induced magnetic state has been demonstrated by the electrical transport study [20]. Figure 9a, b show the temperature-dependent magneto-transport results for fully strained (1.7 nm) and anisotropically strain-relaxed (9.1 nm) RuO_2_ films, respectively. Non-linear behavior is observed only in the fully strained film below 15 K, whereas the resistivity of the strain-relaxed film varies linearly with the magnetic field. This indicates the emergence of a magnetic state in RuO_2_ thin films under strain. The authors also ruled out the possibility of multi-carrier conduction. The strain-induced magnetic state was explained by the Stoner criterion, arising from the strong modulation of the 4d_x_^2^_−__y_^2^ orbital near the Fermi level. Figure 9c presents the strain (ε)-dependent density of states (DOS), where ε = 1 corresponds to the fully strained state, calculated with Hubbard U = 0. As ε increases, the narrow peak near − 0.5 eV systematically shifts toward the Fermi level, which can induce an itinerant AFM state as described by the Stoner criterion.

Optical second harmonic generation (SHG) and magneto-optics measurements on MBE-grown fully strained RuO_2_/TiO_2_ (110) film further support broken time-reversal symmetries accompanied by inversion symmetry breaking, suggesting the emergence of strain-induced polar metallic AM state [48, 56] (Fig. 9d). Furthermore, spin-ARPES results at 10 K on the fully-strained RuO_2_ further confirm the coexistence of both electrical polarization and AM, the spin-split bands in momentum space display both mirror-odd (Rashba-type, from inversion symmetry breaking) and mirror-even (AM) spin textures in epitaxially strained RuO_2_ films [13]. The distinct spin orientations observed in the two experiments are likely due to differences in measurement temperature and methodology between SHG and spin-ARPES, yet both observations are consistent with the breaking of time-reversal symmetry. It might reflect also the possible spin canting in the strained RuO_2_ film, suggesting the need for further investigations to reveal the spatially resolved magnetic state. Despite the distinct spin orientations observed experimentally, both corresponding magnetic point groups (*m*′*m*′2 and *m*′*m*2′) are classified as altermagnets [64].

These theoretical and experimental results for hybrid MBE-grown RuO_2_ thin films grown on TiO_2_ substrate highlight the complex, thickness-dependent strain relaxation behavior and emphasize the critical role of strain in determining not only the electronic states of Ru 4d orbitals but also the magnetic properties of RuO_2_. Therefore, precise characterization of strain states in RuO_2_ is essential for exploring its potential AM ground state.

## Conclusion and outlook

Rutile RuO_2_ has rapidly emerged as a promising AM candidate, generating significant excitement—and ongoing debate—within the condensed matter and spintronics communities. Its *d*-wave symmetry and high electrical conductivity inspire intriguing spin-torque effects and large tunneling magnetoresistance, in contrast to established *g*-wave-symmetry altermagnetic candidates such as MnTe [65, 66] and CrSb [33]. Although the precise magnetic ground state remains under discussion, particularly whether the observed symmetry breaking truly corresponds to AM, growing evidence indicates that epitaxial strain and nanoscale defects play a central role in stabilizing AM signatures in RuO_2_ thin films. These observations highlight the urgent need for defect-managed bulk crystals and epitaxially engineered films to reliably access and interrogate symmetry-protected magnetic states. The studies conducted so far also make clear that meaningful progress in this field requires impeccably characterized materials—structurally, compositionally, and defect-wise—before reaching any consensus on the intrinsic magnetic behavior of RuO_2_. In this respect, a systematic spin-ARPES study that directly probes altermagnetic spin splitting in RuO_2_ would serve as a “smoking gun”, provided that the strain states in RuO_2_ films are carefully controlled and characterized.

Beyond the need for improved materials quality, RuO_2_ possesses intrinsic *advantages* that make it an exceptional platform for exploring and tuning AM physics. Its moderate electronic correlation strength enables fine control of its electronic structure through chemical doping, epitaxial strain, and dimensionality engineering such as ultrathin films and heterointerfaces. This tunability provides a direct experimental route to manipulate the interplay among crystal symmetry, spin–orbit coupling, and exchange interactions that underlie AM order. Recent ab initio and spectroscopic studies have already shown that modest lattice distortions or carrier doping can reshape the spin-split Fermi surfaces and potentially enhance AM [15, 20], pointing toward feasible pathways for achieving controllable AM functionalities.

Experimentally, a wide range of spin-dependent transport phenomena have now been reported in RuO_2_, collectively building the case for its emerging role as a metallic AM. Pronounced magnetoresistance, AHE, spin Hall effect, and spin–orbit torque measurements all reveal strong spin-momentum interplay [14–20, 22–31]. Particularly compelling are TMR studies in RuO_2_-based junctions—with and without conventional FM electrodes—which provide direct evidence for spin-polarized electronic states and demonstrate that RuO_2_ can induce spin-dependent tunneling purely through its symmetry-driven AM band structure [22, 23].

Taken together, RuO_2_’s high metallicity, strong electronic structure tunability, and emerging TMR response establish it as a leading candidate for next-generation AM spintronics. Its excellent conductivity enables efficient charge-to-spin conversion and easy integration with existing oxide and metallic device architectures. Moving forward, systematic exploration of TMR and related transport phenomena under carefully controlled strain, doping, and dimensional confinement will be essential for correlating structural and electronic parameters with AM signatures. Such studies could ultimately position RuO_2_ as the prototypical material where symmetry-driven magnetism translates into practical, energy-efficient spintronic devices operating at room temperature.

## Data Availability

All data supporting the results within this paper are available from the corresponding author upon reasonable request.
